# The Fishes of the United States

**Published:** 1853-08

**Authors:** 


### THE FISHES OF THE UNITED STATES.

We lay the following letter before our readers in the hope that among those in Kentucky there may be some who will be disposed to co-operate in the work alluded to by Prof. Agassiz. It is a work in which every lover of science in our country must feel an interest. We trust, by means of this circular, that we shall be able to collect some information useful to the great naturalist; and have to beg the favor of any of our friends who can give assistance themselves in collecting the fishes of Kentucky, or can refer us to others who will engage in it, to communicate with us, or directly with Prof. Agassiz, on the subject.

Cambridge, 14th July, 1853

*Dear Sir:*

The lively interest you have taken for so many years in the progress of science in this country may be my apology for asking your co-operation in an investigation in which I am deeply engaged.

Perhaps I may also trust to your kindness towards me, and assure you I shall value highly any assistance you may be willing to render me on this occasion. For years I have been collecting materials for a general Natural History of the Fishes of the United States, in which I should like not only to give as complete a description as can be drawn up from preserved specimens of all the species found in our waters, but also a minute account of their geographical distribution, which it is high time to study now, before increased navigation has modified it extensively. It becomes, therefore, necessary that I should obtain separate collections of the fishes, shells, and crawfishes, which I mean to include all in my investigations, from every watercourse in the United States. This is such a gigantic undertaking for a private gentleman, that I trust scientific men may assist me in different parts of the country, whilst I should be happy to repay any expense they incur in making such collections, if I only can have them brought together.

In this respect, the State of Kentucky is one of the most important of the Union, not only on account of the many rivers which pass through its territory, but also because it is one of the few States the fishes of which have been described by former observers, especially by Rafinesque in its Ichthyologia Ohiensis, so that a special knowledge of all his original types is a matter of primary importance for any one, who would compare with each other, the fishes of the different rivers of the West. Would it be asking too much of you to inform me of the persons in different parts of your State, who might be applied to, to make such collections for me. From the Ohio river I should want three distinct collections, one made anywhere between the junction of the Big Sandy river with the Ohio at Maysville, another about Louisville, and a third anywhere between the mouth of Green river and that of the Cumberland rivrer. From the tributaries of the Ohio I want a collection from the Big Sandy river, another from the Licking, another from the Kentucky river, even two if possible, one from its headwaters and another from its lower course above its junction with the Ohio, another from Salt river, if possible two of Green river, one above and another below the Mammoth Cave, one from the headwaters of the Cumberland and another from its lower course, as also one from the lower course of the Tennessee river. Indeed, it might be desirable to obtain even more collections from all the different creeks forming by their reunion these different rivers, were it possible to obtain a sufficient corps of volunteers for such a purpose. However, there is no sort of difficulty in making such collections; all that is required is a keg of alcohol, one head of which is taken off and used as a cover, until the keg is full. In packing it to send off, it is only necessary to see that the fishes are placed close together in layers like herrings, filling the barrel to the very rim, to prevent any jarring and rubbing of its contents; perhaps an occasional layer of paper or cotton cloth between the layers of fishes will assist in keeping them in good condition. After heading again the keg, it is to be filled through the bung hole, or a hole in one head with alcohol of about 80 spec. grav., or rather somewhat stronger. Too much alcohol is injurious, inasmuch as it contracts the fishes and renders them stiff. The alcohol first used to receive the specimens may be employed to pack them, after strengthening it somewhat with a purer article. Could you not make an appeal in my behalf to your people, and mention to me the names of gentlemen living upon your different watercourses in appropriate situations, who might be induced to collect in that way, to aid me in the preparation of a work, which I should like to render so valuable that it would appear as a national standard work. You see that it would require no knowledge of the fishes or shells themselves to make such collections, but only good will, and an opportunity to ask from fishermen specimens of all the fishes of their respective localities. I have found, also, that boys take readily hold of such matters, indeed there are many species which may better be obtained from them than from fishermen, who generally value only the marketable kinds, whilst we naturalists esteem a minnow as highly as a trout, at least for our collections. Of the small kinds of fishes I should like as many as twelve specimens, four to six of those of medium size, and one or two of the larger ones

I would naturally ask first, whether there is not in Louisville some young naturalist among your students, who would take hold of this subject, and make a thorough collection of all the fishes of your immediate vicinity, or could you see yourself to it? I never regretted more than now that circumstances have not yet allowed me to visit your State and to make a stay in Louisville; but I trust it will be one of my first excursions.

